# Structural Analysis and Anticoagulant Activity of Fucosylated Glycosaminoglycan from Sea Cucumber *Phyllophorus proteus*

**DOI:** 10.3390/foods13182889

**Published:** 2024-09-12

**Authors:** Jingwen Liu, Lihua Geng, Jing Wang, Yang Yue, Ning Wu, Quanbin Zhang

**Affiliations:** 1 CAS and Shandong Province Key Laboratory of Experimental Marine Biology, Center for Ocean Mega-Science, Institute of Oceanology, Chinese Academy of Sciences, Qingdao 266071, China; ljw_990304@163.com (J.L.); lhgeng@qdio.ac.cn (L.G.); jingwang@qdio.ac.cn (J.W.); yueyang@qdio.ac.cn (Y.Y.); wuning@qdio.ac.cn (N.W.); 2 Laboratory for Marine Biology and Biotechnology, Qingdao Marine Science and Technology Center, Qingdao 266071, China; 3 University of Chinese Academy of Sciences, Beijing 101408, China

**Keywords:** anticoagulation, fucosylated glycosaminogiycan, *Phyllophorus proteus*

## Abstract

*Phyllophorus proteus* is a low-value sea cucumber from Indonesia and other tropical peripheral waters. In this study, a fucosylated glycosaminoglycan (FG) was extracted from *P. proteus*. It consists of GlcA, GalNAc, and Fuc, with a molecular weight of 67.1 kDa. The degraded FG (dFG) was prepared by β-elimination. Structural analysis revealed that the main chain of dFG was composed of GalNAc and GlcA, linked alternately by β1,3 and β1,4 glycosidic bonds. The sulfate group was located at the 4 and 6 positions of GalNAc. Fuc was attached to the 3 position of GlcA by an α1,3 glycosidic bond, and the side chain of Fuc exhibited various sulfate substitutions. FG significantly prolonged the coagulation time of APTT, PT, TT, and FIB, surpassing the effect of LMWH, thereby demonstrating its ability to exert anticoagulant effects in both the endogenous and exogenous coagulation pathways. Conversely, dFG had no significant effect on the clotting time of PT, suggesting its lack of impact on the intrinsic coagulation pathway. This study elucidates the structural properties and potent anticoagulant activities of fucosylated glycosaminoglycan from *P. proteus*.

## 1. Introduction

Sea cucumber, belonging to the phylum Echinodermata, has been regarded as a traditional tonic in China, with potential for daily health care and medicinal use [1]. The main edible and medicinal parts of sea cucumber are the body wall, which contains a rich array of nutrients, including polysaccharides, saponins, and fatty acids. Among these, sea cucumber polysaccharides are important bioactive substances [2]. Sulfated polysaccharides from sea cucumber are mainly classified into two types: fucosylated glycosaminoglycans (FG) and fucan sulfate (FS) [3]. Fucosylated glycosaminoglycans are primarily found in sea cucumbers and possess unique structural properties and biological activities [4,5,6,7,8].

The structures of fucosylated glycosaminoglycans are relatively regular, but there are differences in their linkage and sulfate substitution patterns. Fucosylated glycosaminoglycans from various sources of sea cucumbers exhibit noticeable structural diversity [9]. In 1988, Brazilian scholars Mourao et al. [10] first reported FG from *Ludwigothurea grisea* displayed a similar main chain to chondroitin sulfate. The main difference between FG from *Ludwigothurea grisea* and chondroitin sulfate from common mammalian sources is the presence of α1,3 glycosidically linked fucose side chains at the 3 position of the glucuronic acid of the main chain of FG. There are several specific structural differences between FGs from different sea cucumber sources, including the substitution site of sulfation and fucose and their proportions. For instance, fucosylated glycosaminoglycan (PmFG) found in *Pattalus mollis* consists of (-4-D-GlcA-β-1,3-D-GalNAc4S6S-β-1-) repeating units constituting the backbone, and its side chain consists of differently sulfated fucose linked by α1,3 glycosidic bonds, including Fuc3S4S, Fuc2S4S, and Fuc4S, with a molar ratio of 2.5:2:1 [11]. The side chains of fucosylated glycosaminoglycans found in *Holothuria mexicana* were Fuc2S4S and Fuc4S attached to the O-3 position of the glucuronic acid [12]. While most of the common FG side chains were monosaccharide, some sea cucumber sources also exhibited disaccharide side chains. FG isolated by Nadezhda E [13] in *Eupentacta fraudatrix* had a disaccharide side chain attached to O-3 of GlcA. FCSsj, extracted from the body wall of the sea cucumber *Stichopus japonicas* [14], revealed the presence of an Fuc2S4Sα (1→3) Fuc4S branch attached to the C3 position of GlcA. The side chains of the majority of sea cucumber-derived FGs were composed of fucose only, with fucose primarily attached to position 3 of GlcA. However, recent studies have shown the presence of GalNAc on some FG side chains and even side chains located at the 6 position of GalNAc. HHFG, extracted from *Holothuria hilla* [15], contained Fuc4S, Fuc3S4S, and Fuc2S4S in a ratio of 1:1:1.5, and in addition, some GalNAc residues in HhFG contained the unusual disaccharide branching at O-6, Fuc4S-(1→2)-Fuc3S4S-(1→). AmFG, obtained from *Acaudina molpadioides* [16], contained an uncommon disaccharide branching GalNAc-α1, 2-Fuc3S4S (15%), which is the first report of this type of structure in a sea cucumber-derived FG. In summary, FGs from sea cucumber sources have a similar chondroitin sulfate main chain structure but display considerable variations in the linkage mode of the side chain, type of sugar, degree of polymerization, and proportion of sulfate substitution.

Besides natural fucosylated glycosaminoglycans from sea cucumbers, in the past decades, low-molecular-weight FG or FG oligosaccharides were prepared by synthetic or semi-synthetic approaches [17]. In 2013, Tamura and co-workers first totally synthetized an FG trisaccharide unit most commonly found in natural FG—4,6-di-O-sulfated-β-GalNAc-(1→4)-[2,4-di-O-sulfated-α-Fuc-(1→3)]-GlcA—[18]. Thereafter, several types of FG oligosaccharides were synthetized. In addition to the total synthesis, semi-synthetic strategies based on GAGs or similar natural polysaccharides as starting materials were also developed to synthetize FG oligosaccharides or low-molecular-weight FG [19]. The total synthesis and/or semi-synthesis of FG oligosaccharides and low-molecular-weight FG would be helpful in better understanding the structure–bioactivity relationships of FG.

Recently, FGs have been reported to exhibit potent anticoagulant activities. These polysaccharides demonstrate excellent anticoagulant effects through the inhibition of prothrombin (FIIa) and factor Xa (FXa) mediated by antithrombin III (ATIII), and it has been established that molecular weight and degree of sulfation greatly impact the anticoagulant and antithrombotic activities of FGs [20]. FG oligosaccharides exhibit excellent anticoagulant activity with lower risks of adverse effects and bleeding [21]. Furthermore, the anticoagulant effect of FGs is closely associated with the presence of sulfated fucose-branched chains [22]. Given their structural variability, understanding the structure–activity relationship of FGs remains a challenge.

*Phyllophorus proteus*, a type of sea cucumber primarily found in Indonesia and other tropical Asian countries, has a high production volume but is rarely consumed locally. Unfortunately, studies exploring the bioactive substances and potential applications of *P. proteus* are scarce. Qin et al. optimized the enzyme-assisted extraction method of polysaccharides from *P. proteus* and demonstrated that these polysaccharides exhibited obvious antioxidant activities [23]. The objective of this study is to investigate the structure and anticoagulant activities of fucosylated glycosaminoglycan from *P. proteus*, with the aim of providing a scientific basis for the valuable application of *P. proteus*.

## 2. Materials and Methods

### 2.1. Preparation of Polysaccharides from P. proteus

Dried *P. proteus* from Indonesia was purchased on www.JD.com on 12 May 2022 and authenticated by an expert of the Institute of Oceanology, Chinese Academy of Sciences. Polysaccharides were extracted by the method of enzyme-alkaline digestion. Dried *P. proteus* was soaked in deionized water overnight, deveined, homogenized, and hydrolyzed with 1% papain at 60 °C for 6 h. NaOH was then added to the final concentration of 0.25 M, and alkaline digestion was carried out at 60 °C for 2 h. The solution was neutralized with HCl, heated at 100 °C for 10 min to inactivate the enzyme, and then centrifuged at 2880× *g* for 20 min. The supernatant was precipitated with 60% (*v*/*v*) ethanol and left at 4 °C overnight. After centrifugation at 2880× *g* for 20 min, the precipitate was dissolved in distilled water, dialyzed (Mw = 3.5 kDa), concentrated, and finally lyophilized to obtain the crude polysaccharide.

The crude polysaccharide was dissolved in distilled water and fractionated on a Sepharose DEAE Fast Flow anion-exchange chromatographic column (2.6 × 100 cm), eluted with gradient elution of increased concentration of NaCl solution from 0 to 2.0 M. The elution was detected by the phenol–sulfuric acid method. Each elution was combined, dialyzed, concentrated, and finally lyophilized. In this process, four fractions corresponding to the NaCl concentrations of 0.4 M, 1.1 M, 1.4 M, and 2.0 M, respectively, were obtained, and the fraction eluted with a 1.1 M NaCl solution was proved to be a fucosylated glycosaminoglycan (FG). The main fractions were further purified on a Sephadex-G100 chromatographic column (GE, Uppsala, Sweden). 

### 2.2. β-Elimination of Fucosylated Glycosaminoglycan

FG was degraded by the method of β-elimination according to the previously reported procedure [24]. In brief, 800 mg of FG was dissolved in 12 mL of water, and benzyl ammonium chloride solution was slowly added to a final concentration of 62.5 mg/mL, centrifuged at 3220× *g* for 15 min, then left at 4 °C overnight. The precipitate was washed with deionized water three times, and each time it was left at 4 °C for 4 h. The precipitate was dried at 40 °C in a vacuum drying oven to obtain the FG-quaternary ammonium salt precipitate, which was dissolved with 10 mL of DMF (200 mg/mL). Approximately 0.43 mL of benzyl chloride was added to the solution and stirred at 35 °C for 25 h. The sodium ethanolate–ethanol solution was added to a final concentration of 0.02 M and stirred for 30 min. An equal volume of saturated NaCl solution was added; after that, anhydrous ethanol was added to a final concentration of 80% (*v*/*v*), left for 6 h, then centrifuged at 3220× *g* for 15 min. The precipitate was dissolved in 120 mL of water, and a 4 M NaOH solution was added to a final concentration of 0.05 M. After reaction for 30 min, NaBH_4_ was added to a final concentration of 0.1 M, and the reaction continued for 30 min. At the end of the reaction, the solution was adjusted to neutral with HCl. The β-elimination product was desalted on Sephadex-G10, concentrated, and lyophilized.

### 2.3. Molecular Weight and Chemical Analysis

The total sugar content of the polysaccharides was analyzed using the phenol–sulfuric acid method [25]. The fucose content was analyzed using the cysteine hydrochloride method [26]. The uronic acid content was analyzed using the carbazole colorimetric method [27]. The protein content of the polysaccharides was analyzed using the BCA kit. The sulfate content was determined by the gelatin-barium chloride colorimetric method [28]. The monosaccharide compositions of the polysaccharides were determined using the precolumn PMP derivation HPLC method [29] Molecular weights of polysaccharides were measured by HPGPC using a Shimudzu LC 20 AT (Shimadzu, Tokyo, Japan)apparatus equipped with a TSK gel PWxl 3000 column (7 μm 7.8 × 300 mm), a differential refractive index (RI) detector, and a diode array detector (DAD). The weight-average molecular mass (Mw) was calculated by Shimudzu LabSolutions GPC software Version 6.80 using a standard curve made by dextran standards.

### 2.4. IR and NMR Spectroscopy

Infrared spectra (IR) were determined on a Nicolet-360 FTIR spectrometer (Thermo Fisher Scientific Inc., Waltham, MA, USA) between 400 and 4000 cm^−1^.

For NMR analysis, FG and dFG were dissolved in 99.9% D_2_O and freeze-dried. Then they were re-dissolved in 99.96% D_2_O. The NMR spectra of ^1^H, ^13^C, COSY, HSQC, HMBC, and TOCSY were recorded at 60 °C on a Bruker 600 MHz spectrometer (Bruker Corporation, Billerica, MA, USA). Deuterated acetone was used as an internal standard.

### 2.5. In Vitro Anticoagulant Assay

Plasma recalcification experiments were performed using FG and dFG at a concentration of 10 μg/mL. Ten microliters of samples and 40 μL of human plasma were added into the 96-well plate, shaken for 20 s, and incubated at 37 °C for 10 min. Then, 50 μL of CaCl_2_ solution at a concentration of 50 μM was quickly added, and the absorbance at 650 nm was measured at 30 s intervals. 

In vitro anticoagulant activity of the activated partial thromboplastin time (APTT, assay kits from Leagene, Beijing, China), thrombin time (TT, assay kits from Leagene, Beijing, China), prothrombin time (PT, assay kits from Leagene, Beijing, China), and fibrinogen time (assay kits from SunBio, Shanghai, China) were measured according to the kits manufacturer’s instructions. Sheep plasma from Shanghai Yuanye Bio-Technology Co., Ltd. (Shanghai, China). was used for these assays. 

### 2.6. Statistical Analysis

All data are presented as the mean ± standard deviation (SD). Individual differences between the means were determined using the *t*-test, and statistically significant differences were considered to be present when *p* < 0.05.

## 3. Results and Discussion

### 3.1. Preparation and Chemical Properties of Polysaccharide Fractions

Polysaccharides were extracted using enzyme-alkaline digestion, resulting in a crude polysaccharide yield of 2.86%. This crude polysaccharide was then subjected to gradient elution on a Sepharose DEAE Fast Flow column chromatography using a linear elution of 0–2.0 M NaCl solution. The elution was detected by the phenol–sulfuric acid method. Each elution was combined, dialyzed, concentrated, and finally lyophilized. In this process, four fractions corresponding to the NaCl concentration of 0.4 M, 1.1 M, 1.4 M, and 2.0 M, respectively, were obtained. Table 1 shows the chemical composition analysis of the four fractions. It is notable that the 0.4 M eluted fraction had very low fucose, sulfate, and uronic contents, indicating it is not a sulfated polysaccharide. Conversely, the 1.4 M and 2.0 M eluted fractions exhibited high fucose content and low uronic acid content, suggesting that these two fractions are fucan sulfates. For the 1.1 M NaCl eluted fraction, the total sugar content, fucose content, and sulfate content were 41.13%, 13.77%, and 24.36%, respectively. The monosaccharide composition analysis revealed that the main monosaccharides present in the 1.1 M eluted fraction were GalN, GlcA, and Fuc. It should be noted that during the analysis, GalNAc lost its acetyl group due to TFA hydrolysis, thus yielding GalN [15,16]. These findings indicate that this fraction is an FG fraction.

**Figure 1:**
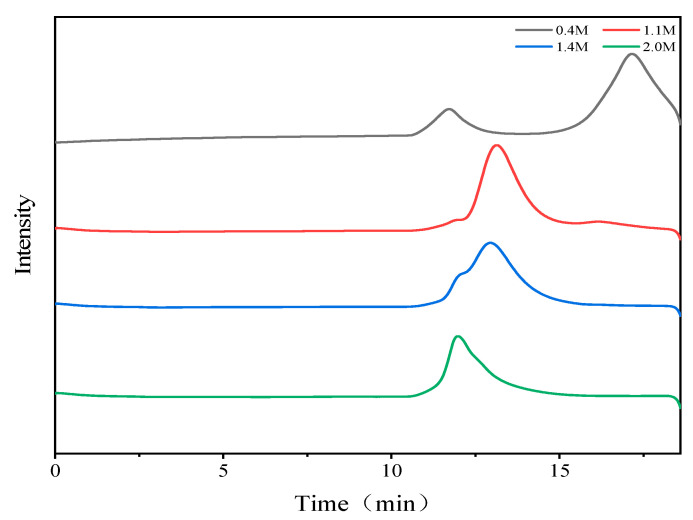
Molecular weight determination of the four polysaccharide fractions by HPGPC.

The molecular weights of the four fractions were determined using HPGPC, and the results are depicted in Figure 1. The weight average molecular weights of the 0.4 M, 1.1 M, 1.4 M, and 2.0 M NaCl eluted fractions were 205.8 kDa, 67.1 kDa, 91.6 kDa, and 137.8 kDa, respectively. While the 1.1 M and 2.0 M eluted fractions showed roughly symmetric elution peaks, the 1.4 M eluted fraction exhibited a shoulder peak corresponding to the 2.0 M eluted fraction.

### 3.2. Purification and Analysis of FG

**Figure 2:**
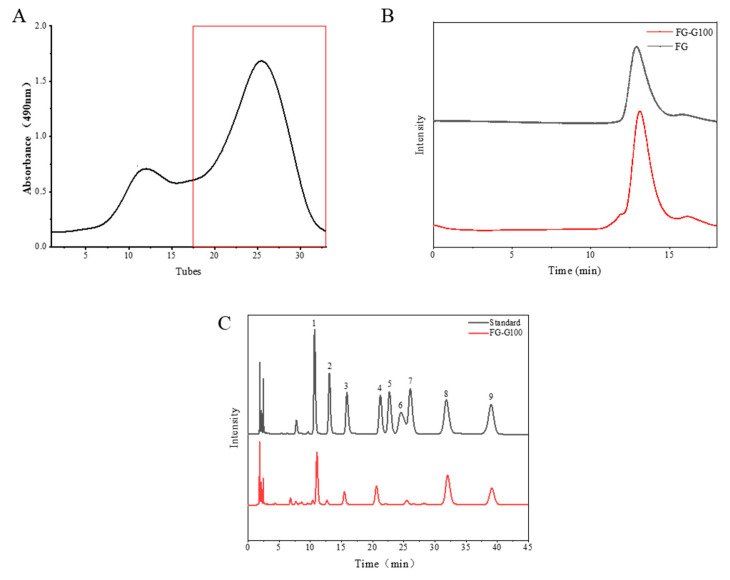
Elution curve (**A**), molecular weight (**B**), and monosaccharide composition (**C**) analysis of FG purified by Sephadex G-100 column chromatography. ((**C**): 1 PMP; 2 GlcN; 3 GlcA; 4 GalN; 5 Glc; 6 GalNAc; 7 Gal; 8 Fuc; 9 Rib).

FG was purified using Sephadex G100 column chromatography, and the elution curve is shown in Figure 2A. High-performance liquid chromatography (HPLC) and monosaccharide composition analysis of the purified FG indicated reduced dispersion in the chromatograms (Figure 2B). The monosaccharide composition analysis revealed that the purified FG (FG-G100) consisted of GalNAc, GlcA, and Fuc (Figure 2C).

**Figure 3:**
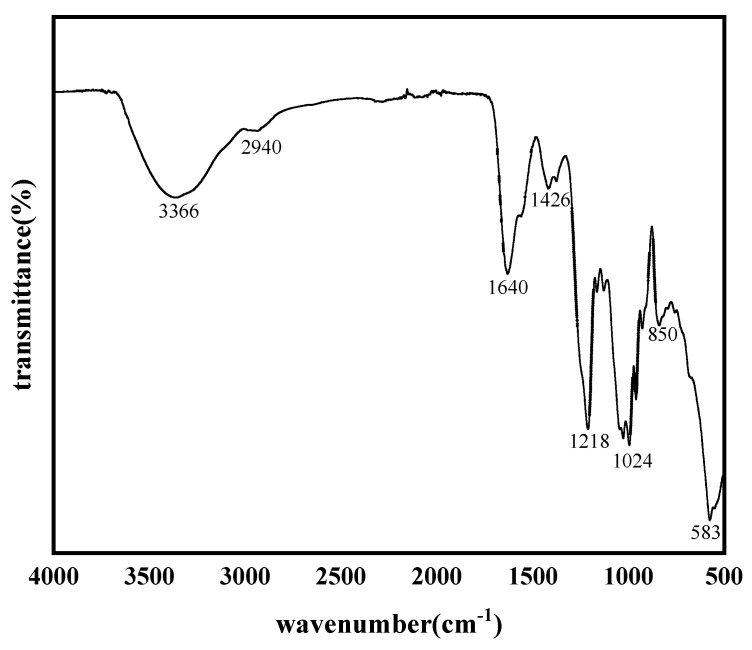
Infrared spectrum of FG (1.1 M elution fraction).

The infrared spectrum of FG is illustrated in Figure 3. The absorption peaks at 3366 and 1024 cm^−1^ correspond to the stretching vibrations of hydroxyl O-H and C-O-C in the glycocyclic ring, respectively. The peak at 2940 cm^−1^ represents the stretching vibration of methylene and methyl C-H. The absorption peaks at 1218 and 850 cm^−1^ indicate the presence of asymmetric stretching vibration of S=O and the bending vibration of C-O-S. The stretching vibration of the C=O double bond of acetylgalactosamine and glucuronic acid is depicted at 1640 cm^−1^.

**Figure 4:**
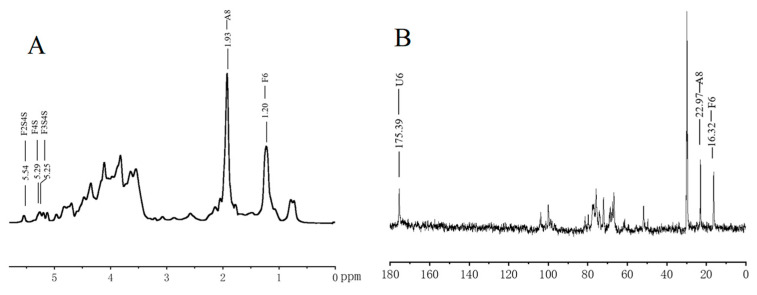
^1^H-NMR (**A**) and ^13^C-NMR (**B**) spectra of FG.

The structural properties of FG were analyzed using NMR. The signal at 1.20 ppm in the ^1^H spectrum (Figure 4A) corresponded to the methyl proton of Fuc, the signal at 1.93 ppm represented the methyl proton on the acetylamino group of GalNAc, and the signals at 5.0–5.5 ppm indicated the anomeric proton on fucose with different sulfation patterns. Due to severe signal overlapping, the signals between 3.0 and 5.0 ppm were difficult to resolve. The ^13^C-NMR spectrum results (Figure 4B) demonstrated that the signal at 16.32 ppm indicated the methyl carbon of Fuc, 22.97 ppm corresponded to the methyl carbon on the acetylamino group of GalNAc, and 175.39 ppm represented the carboxyl of GlcA. Similar to the ^1^H spectrum, the signals between 40 and 120 ppm were challenging to resolve due to the large molecular mass and severe overlap.

### 3.3. Preparation and Structural Analysis of dFG

**Figure 5:**
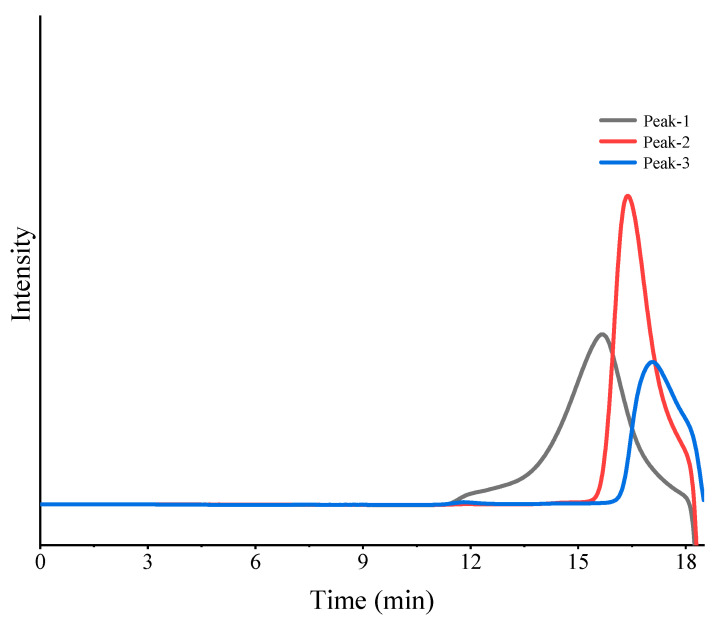
Molecular weight measurement by HPLC of dFG-P6 elution fractions.

The large molecular mass of FG hindered its structural elucidation. To address this, FG was degraded by β-elimination. Subsequently, low-molecular-weight products dFG were obtained and repeatedly purified using Sephadex G100 and Bio-Gel P-6 column chromatography. Three different fractions of dFG were acquired, with molecular weights of 19.3 kDa, 5.0 kDa, and 3.1 kDa, respectively (Figure 5).

**Figure 6:**
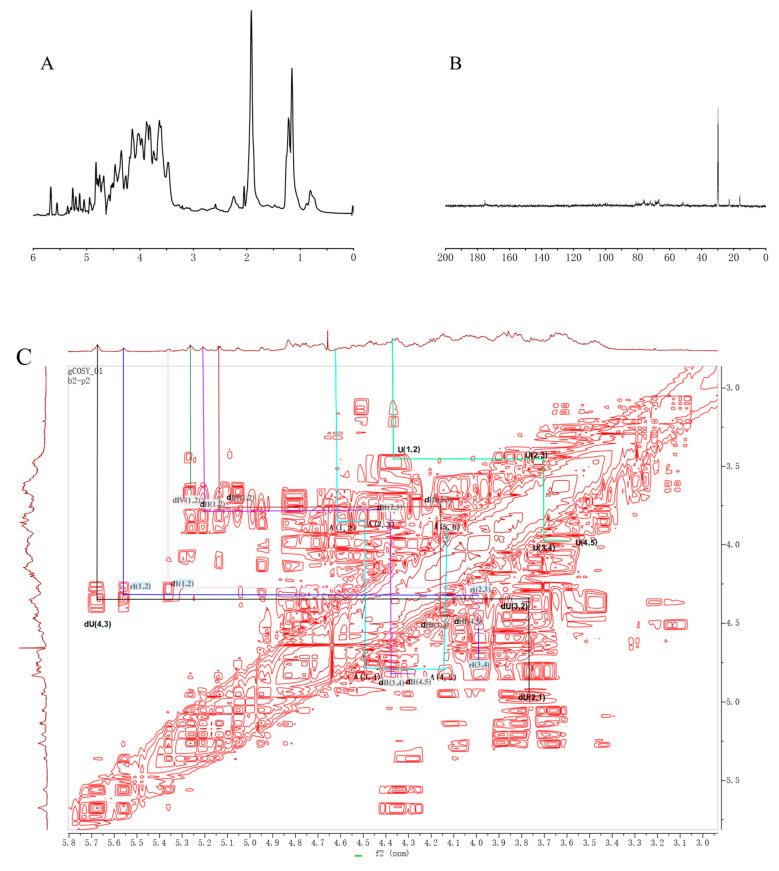

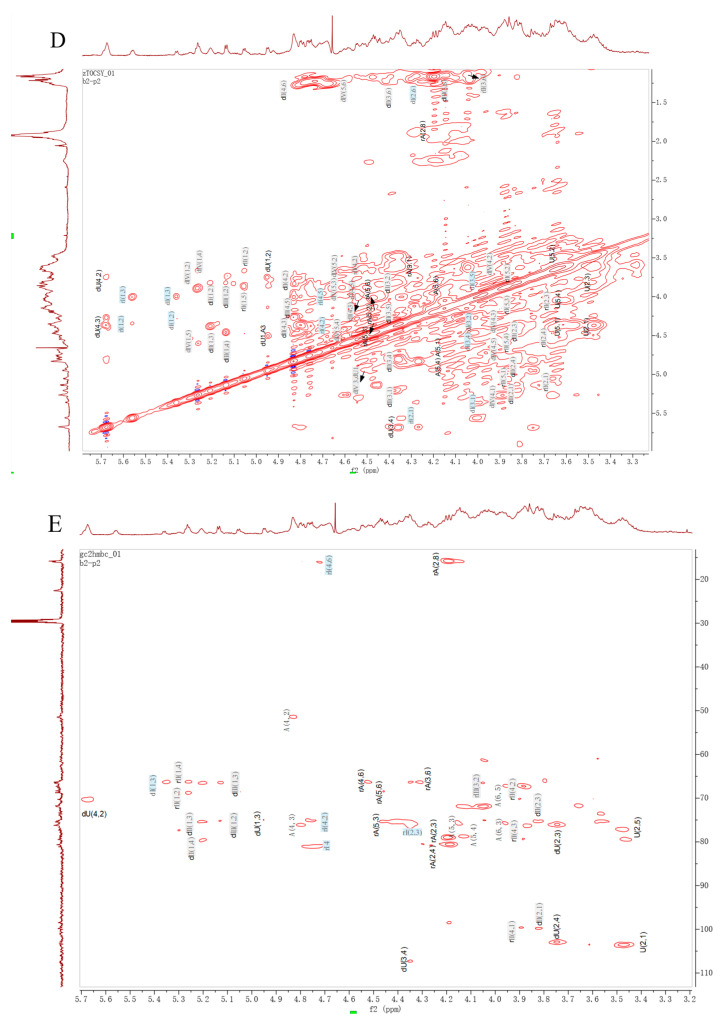

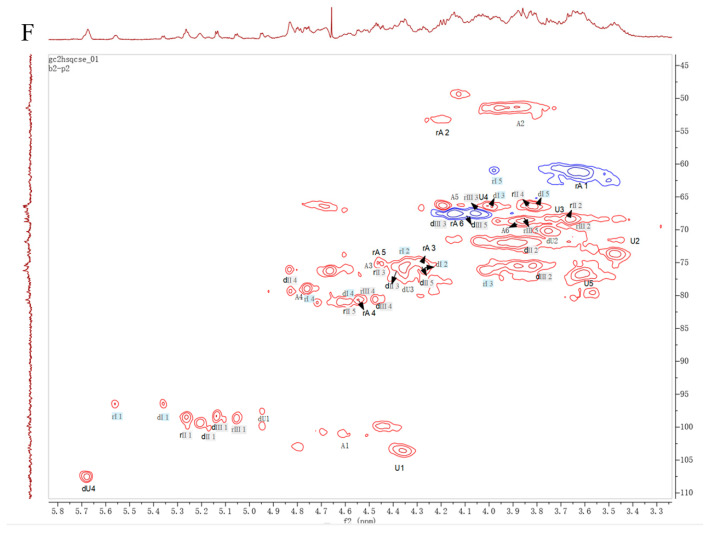
1D and 2D-NMR spectra of dFG. ^1^H NMR (**A**); ^13^C NMR (**B**); ^1^H-^1^H COSY (**C**); ^1^H-^1^H TOCSY (**D**); ^1^H-^13^C HMBC (**E**); ^1^H-^13^C HSQC (**F**).

**Figure 7:**
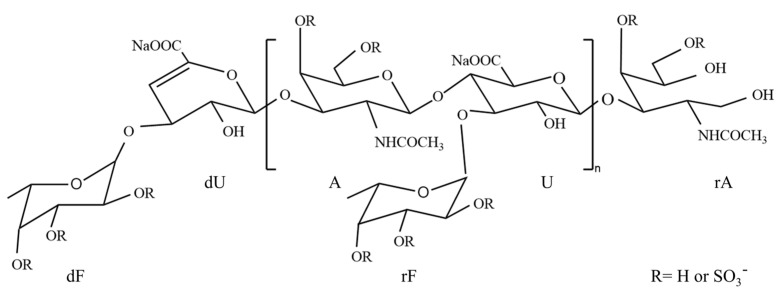
The structure of dFG.

The 5.0 kDa dFG fraction was analyzed using NMR (Figure 6). The signal at 5.68/107.53 ppm represented the characteristic signal of the unsaturated hydrogen/carbon of the non-reducing end of glucuronic acid (dU) produced by β-elimination. The proton/carbon at the 1 position of the unsaturated glucuronic acid (dU) was detected at 4.95/97.71 ppm. The signals at 5.36, 5.26, 5.21, and 5.13 ppm were attributed to the hydrogen signals of the anomeric protons of F2S4S, F3S, F3S4S, and F4S, respectively [29]. Additionally, the signals at 1.87 and 1.92 ppm represented the methyl hydrogen on the acetylamino group of acetamido-galactose. The other signals corresponding to the sugar group were further confirmed using ^1^H-^1^H COSY, ^1^H-^1^H TOCSY, ^1^H-^13^C HMBC, and ^1^H-^13^C HSQC. The HMBC correlation spectra indicated the presence of correlation signals between the H-1 of GlcA and the C-3 of GalNAc, indicating that GlcA is attached to the C-3 position of GalNAc4S6S by a β1,3 glycosidic bond. The correlation between the H-1 of Fuc and the C-3 position of GlcA suggested that Fuc is attached to the C-3 of GlcA by an α1,3 glycosidic bond. Combining all the structural information, a possible structure of dFG was displayed in Figure 7. The dFG from *P. proteus* was composed of GlcA and GalNAc alternately linked by β1,3 and β1,4 glycosidic bonds. The sulfate groups were located at the 4 and 6 positions of GalNAc [15], while Fuc was linked to the 3 positions of GlcA by α1,3 glycosidic bonds. Various sulfate substitutions were present in the side chain of the Fuc glycosidic bond. Specific signal attributions are shown in Table 2.

### 3.4. Anticoagulant Activities

**Figure 8:**
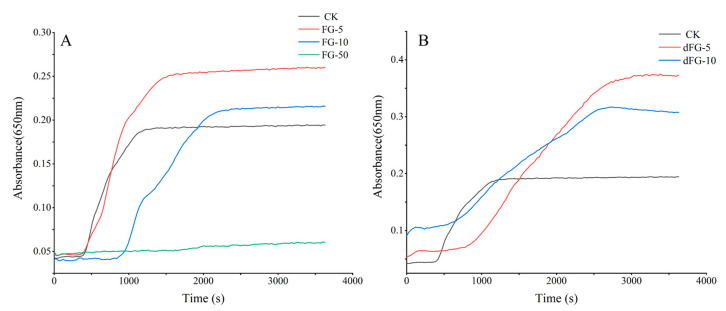
Plasma recalcitrance experiments of FG (**A**) and dFG (**B**).

The anticoagulant activities of FG and dFG were initially assessed by measuring their effects on the recalcification time of sheep plasma. At a concentration of 10 μg/mL, both FG and dFG were able to delay the coagulation time of plasma, reduce the rate of plasma coagulation, and showed significant anticoagulant activity (Figure 8). FG did not exhibit significant anticoagulant activity at 5 μg/mL, while at a concentration of 50 μg/mL, it kept the plasma uncoagulated during the recording time, demonstrating highly significant anticoagulant activity. On the other hand, dFG showed anticoagulant activity at 5 μg/mL and 10 μg/mL, with the activity increasing with an increase in concentration.

**Figure 9:**
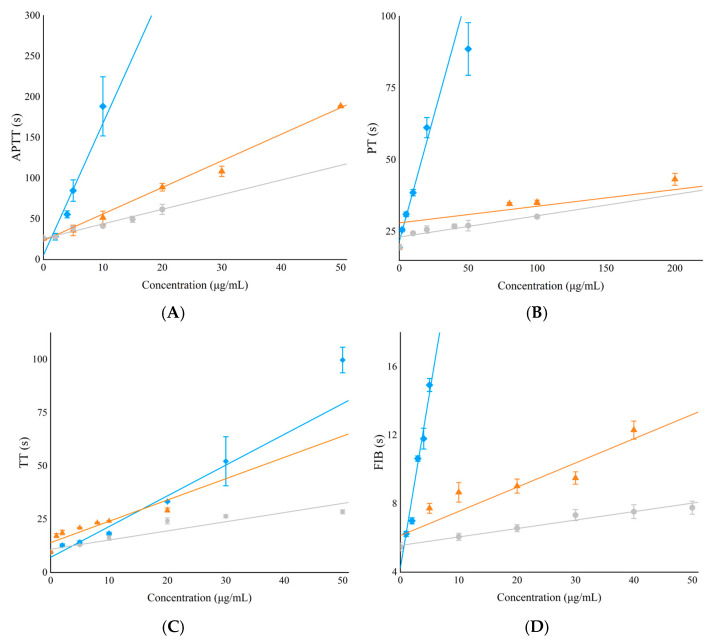
In vitro anticoagulant assay of FG (blue), dFG (orange) and LMWF (grey). (**A**) APTT, (**B**) PT, (**C**) TT, (**D**) FIB.

The APTT assay was employed to determine the effects on intrinsic coagulation pathways. A prolonged APTT indicates the inhibition of intrinsic factors and/or common pathways. PT evaluates extrinsic coagulation pathways while TT represents common coagulation pathways. The in vitro anticoagulant activity of FG and dFG was evaluated using coagulation assays such as APTT, PT, TT, and FIB and compared with low-molecular-weight heparin (LMWH). The results (Figure 9, Table 3) demonstrated that FG displayed significant anticoagulant activity, significantly prolonging the APTT, PT, TT, and FIB clotting times. The concentrations required for FG to double the APTT, PT, TT, and FIB were 4.51, 10.85, 8.03, and 3.36 μg/mL, respectively, whereas for LMWH, the concentrations required were 32.87, 203.17, 49.99, and 112.18 μg/mL. It is evident that FG required much lower concentrations than LMWH to double the duration of APTT, PT, TT, and FIB, indicating a significant anticoagulant activity. The dFG fraction significantly prolonged the coagulation time of APTT, TT, and FIB. The concentrations of dFG required to double the time of APTT, PT, TT, and FIB were 18.78, 192.88, 17.40, and 33.45 μg/mL, respectively. These results indicate that dFG can affect the intrinsic coagulation pathway and the common coagulation pathway but not the extrinsic coagulation pathway, consistent with previous reports on dFG in other species [12].

## 4. Conclusions

In this study, a fucosylated glycosaminoglycan (FG) was extracted from *Phyllophorus proteus*, and its structure was elucidated by NMR analysis of its β-elimination product. FG from *P. proteus* has typical structural properties of fucosylated glycosaminoglycan. The main chain of FG was composed of GlcA and GalNAc, linked alternately by β1,3 and β1,4 glycosidic bonds. The sulfate group was located at the 4 and 6 positions of GalNAc. Fuc was linked to the 3 position of GlcA by an α1,3 glycosidic bond, and the side chain of Fuc exhibited various sulfate substitutions. FG has potent anticoagulant activities, and its activity is greatly related to its molecular weight. FG significantly prolonged the coagulation time of APTT, PT, TT, and FIB, surpassing the effect of LMWH, thereby demonstrating its ability to exert anticoagulant effects in both the endogenous and exogenous coagulation pathways. Conversely, the low-molecular-weight dFG had no significant effect on the clotting time of PT, suggesting its lack of impact on the intrinsic coagulation pathway. This study elucidates the structural properties and potent anticoagulant activities of fucosylated glycosaminoglycan from *P. proteus*. The results suggest that decreasing the molecular weight of FG can enhance the selective effect on specific coagulation pathways, thus might reduce the risk of bleeding while maintaining better anticoagulant activity, but the specific relationship between molecular weight and anticoagulant activity is not clear, and more in-depth studies are needed.

## Figures and Tables

**Table 1:** Chemical composition (%) of the four elution fractions of polysaccharides from *P. proteus*.

Chemical Composition (%)	0.4 M	1.1 M	1.4 M	2.0 M
Total sugar %	25.37	46.63	62.68	62.37
Fucose %	0.25	13.77	23.01	25.32
Uronic acid %	1.45	8.07	5.58	2.90
Sulfate %	0.00	24.36	19.27	14.47
Protein %	30.61	3.41	1.93	2.20

**Table 2:** NMR signal attribution of dFG.

dU	H	δ (ppm)	C	δ (ppm)	rA	H	δ (ppm)	C	δ (ppm)
	H1	4.95	C1	97.71		H1	3.61/3.65	C1	61.25
	H2	3.76	C2	70.30		H2	4.20	C2	53.21
	H3	4.36	C3	76.29		H3	4.29	C3	75.32
	H4	5.68	C4	107.53		H4	4.54	C4	80.72
			C5	146.10		H5	4.47	C5	75.09
			C6	175.33		H6/6′	4.13/4.15	C6	67.62
								C7	178.07
						H8	1.87	C8	26.61
U	H	δ (ppm)	C	δ (ppm)	A	H	δ (ppm)	C	δ (ppm)
	H1	4.37	C1	103.87		H1	4.61	C1	101.06
	H2	3.45	C2	71.74		H2	3.87	C2	51.44
	H3	3.71	C3	68.47		H3	4.50	C3	75.32
	H4	4.00	C4	66.38		H4	4.80	C4	79.31
	H5	3.61	C5	76.63		H5	4.14	C5	67.62
			C6	174.70		H6/6′	3.91/3.96	C6	68.81
								C7	175.77
						H8	1.92	C8	22.59
dI (F2S4S)	H	δ (ppm)	C	δ (ppm)	dII (F3S4S)	H	Δ (ppm)	C	δ (ppm)
	H1	5.36	C1	96.60		H1	5.21	C1	99.28
	H2	4.29	C2	75.32		H2	3.83	C2	71.87
	H3	4.00	C3	66.30		H3	4.39	C3	75.75
	H4	4.56	C4	80.72		H4	4.83	C4	76.09
	H5			66.38		H5	4.27	C5	75.32
	H6			15.87		H6	1.27	C6	16.04
dIII (F4S)	H	δ (ppm)	C	δ (ppm)	dIV (F3S)	H	δ (ppm)	C	δ (ppm)
	H1	5.13	C1	98.51		H1	5.26	C1	98.61
	H2	3.81	C2	75.59		H2	3.68	C2	68.39
	H3	4.15	C3	67.62		H3	4.50	C3	75.32
	H4	4.48	C4	80.70		H4	3.88	C4	66.34
	H5	4.10	C5	67.29		H5	4.60	C5	80.71
	H6	1.18	C6	16.04		H6	1.21	C6	16.06
rI (F2S4S)	H	δ (ppm)	C	δ (ppm)	rII (F3S4S)	H	δ (ppm)	C	δ (ppm)
	H1	5.56	C1	96.56		H1	5.05	C1	98.72
	H2	4.35	C2	75.81		H2	3.67	C2	68.39
	H3	4.01	C3	76.29		H3	4.05	C3	67.55
	H4	4.75	C4	79.11		H4	4.54	C4	80.63
	H5	3.97	C5	61.05		H5	3.86	C5	68.69
	H6	1.14	C6	14.49		H6	1.11	C6	15.71

**Table 3:** Concentrations required for doubling APTT, PT, TT, and FIB.

Samples	APTT (μg/mL)	PT (μg/mL)	TT (μg/mL)	FIB (μg/mL)
FG	4.51	10.85	8.03	3.36
dFG	18.78	192.88	17.40	33.45
LMWH	32.87	203.17	49.99	112.18

## Data Availability

The original contributions presented in the study are included in the article, further inquiries can be directed to the corresponding author.

## References

[1] Gu X., Bai G., Liu L., Zhao Q., Li Y. (2023). Research progress on structural characterization of sea cucumber polysaccharide. Chin. J. Mar. Drugs.

[2] Kang J., Jia X., Wang N., Xiao M., Song S., Wu S., Li Z., Wang S., Cui S.W., Guo Q. (2022). Insights into the structure-bioactivity relationships of marine sulfated polysaccharides: A review. Food Hydrocoll..

[3] Martins A., Alves C., Silva J., Pinteus S., Gaspar H., Pedrosa R. (2023). Sulfated Polysaccharides from Macroalgae—A Simple Roadmap for Chemical Characterization. Polymers.

[4] Chen S., Hu Y., Ye X., Li G., Yu G., Xue C., Chai W. (2012). Sequence determination and anticoagulant and antithrombotic activities of a novel sulfated fucan isolated from the sea cucumber *Isostichopus badionotus*. Biochim. Biophys. Acta (BBA)—Gen. Subj..

[5] Lan D., Zhang J., Shang X., Yu L., Xu C., Wang P., Cui L., Cheng N., Sun H., Ran J. (2023). Branch distribution pattern and anticoagulant activity of a fucosylated chondroitin sulfate from *Phyllophorella kohkutiensis*. Carbohydr. Polym..

[6] Li C., Niu Q., Li S., Zhang X., Liu C., Cai C., Li G., Yu G. (2020). Fucoidan from sea cucumber *Holothuria polii:* Structural elucidation and stimulation of hematopoietic activity. Int. J. Biol. Macromol..

[7] Li Q., Cai C., Chang Y., Zhang F., Linhardt R.J., Xue C., Li G., Yu G. (2018). A novel structural fucosylated chondroitin sulfate from *Holothuria mexicana* and its effects on growth factors binding and anticoagulation. Carbohydr. Polym..

[8] Li R., Yu H., Yue Y., Liu S., Xing R., Chen X., Li P. (2016). Sulfated polysaccharides with antioxidant and anticoagulant activity from the sea cucumber *Holothuria fuscogliva*. Chin. J. Oceanol. Limnol..

[9] Matsuhiro B., Osorio-Román I.O., Torres R. (2012). Vibrational spectroscopy characterization and anticoagulant activity of a sulfated polysaccharide from sea cucumber *Athyonidium chilensis*. Carbohydr. Polym..

[10] Vieira R.P., Mourao P.A. (1988). Occurrence of a unique fucose-branched chondroitin sulfate in the body wall of a sea cucumber. J. Biol. Chem..

[11] Zheng W., Zhou L., Lin L., Cai Y., Sun H., Zhao L., Gao N., Yin R., Zhao J. (2019). Physicochemical Characteristics and Anticoagulant Activities of the Polysaccharides from Sea Cucumber *Pattalus mollis*. Mar. Drugs.

[12] Li S., Zhong W., Pan Y., Lin L., Cai Y., Mao H., Zhang T., Li S., Chen R., Zhou L. (2021). Structural characterization and anticoagulant analysis of the novel branched fucosylated glycosaminoglycan from sea cucumber *Holothuria nobilis*. Carbohydr. Polym..

[13] Ustyuzhanina N.E., Bilan M.I., Dmitrenok A.S., Nifantiev N.E., Usov A.I. (2017). Two fucosylated chondroitin sulfates from the sea cucumber *Eupentacta fraudatrix*. Carbohydr. Polym..

[14] Gong P., Li Q., Wu Y., Lu W.Y., Zeng J., Li H. (2021). Structural elucidation and antidiabetic activity of fucosylated chondroitin sulfate from sea cucumber *Stichopus japonicas*. Carbohydr. Polym..

[15] Ustyuzhanina N.E., Bilan M.I., Dmitrenok A.S., Silchenko A.S., Grebnev B.B., Stonik V.A., Nifantiev N.E., Usov A.I. (2020). Fucosylated Chondroitin Sulfates from the Sea Cucumbers *Paracaudina chilensis* and *Holothuria hilla*: Structures and Anticoagulant Activity. Mar. Drugs.

[16] Mao H., Cai Y., Li S., Sun H., Lin L., Pan Y., Yang W., He Z., Chen R., Zhou L. (2020). A new fucosylated glycosaminoglycan containing disaccharide branches from *Acaudina molpadioides*: Unusual structure and anti-intrinsic tenase activity. Carbohydr. Polym..

[17] Vessella G., Traboni S., Laezza A., Iadonisi A., Bedini E. (2020). (Semi)-synthetic fucosylated chondroitin sulfate oligo- and polysaccharides. Mar. Drugs.

[18] Tamura J., Tanaka H., Nakamura A., Takeda N. (2013). Synthesis of β-d-GalNAc(4,6-diS)(1–4)[α-l-Fuc(2,4-diS)(1–3)]-β-d-GlcA, a novel trisaccharide unit of chondroitin sulfate with a fucose branch. Tetrahedron Lett..

[19] Zhang X., Liu H., Lin L., Yao W., Zhao J., Wu M., Li Z. (2018). Synthesis of fucosylated chondroitin sulfate nonasaccharide as a novel anticoagulant targeting intrinsic factor Xase complex. Angew. Chem.-Int. Ed..

[20] Liu X., Liu Y., Hao J., Zhao X., Lang Y., Fan F., Cai C., Li G., Zhang L., Yu G. (2016). In vivo anti-cancer mechanism of low-molecular-weight fucosylated chondroitin sulfate (LFCS) from sea cucumber *Cucumaria frondosa*. Molecules.

[21] Zhao L., Wu M., Xiao C., Yang L., Zhou L., Gao N., Li Z., Chen J., Chen J., Liu J. (2015). Discovery of an intrinsic tenase complex inhibitor: Pure nonasaccharide from fucosylated glycosaminoglycan. Proc. Natl. Acad. Sci. USA.

[22] Yang W., Cai Y., Yin R., Lin L., Li Z., Wu M., Zhao J. (2018). Structural analysis and anticoagulant activities of two sulfated polysaccharides from the sea cucumber *Holothuria coluber*. Int. J. Biol. Macromol..

[23] Gao N., Lu F., Xiao C., Yang L., Chen J., Zhou K., Wen D., Li Z., Wu M., Jiang J. (2015). β-eliminative depolymerization of the fucosylated chondroitin sulfate and anticoagulant activities of resulting fragments. Carbohydr. Polym..

[24] Dubois M., Gilles K.A., Hamilton J.K., Rebers P.T., Smith F. (1956). Colorimetric method for determination of sugars and related substances. Anal. Chem..

[25] Gibbons M.N. (1955). The determination of methylpentoses. Analyst.

[26] Bitter T.M., Muir H.M. (1962). A modified uronic acid carbazole reaction. Anal. Biochem..

[27] Kawai Y., Seno N., Anno K. (1969). A modified method for chondrosulfatase assay. Anal. Biochem..

[28] Zhang J., Zhang Q., Wang J., Shi X., Zhang Z. (2009). Analysis of the monosaccharide composition of fucoidan by precolumn derivation HPLC. Chin. J. Oceanol. Limnol..

[29] Chahed L., Balti R., Elhiss S., Bouchemal N., Ajzenberg N., Ollivier V., Chaubet F., Maaroufi R.M., Mansour M.B. (2020). Anticoagulant activity of fucosylated chondroitin sulfate isolated from *Cucumaria syracusana*. Process Biochem..

